# Case Report: Successful topical simvastatin therapy in a 2-year-old girl with keratin 16-associated palmoplantar epidermal differentiation disorder

**DOI:** 10.3389/fped.2026.1891570

**Published:** 2026-07-02

**Authors:** Qingmei Zhong, Ying Zhang, Zhen Feng, Jinyan Zhang, Borui Chen

**Affiliations:** 1 Department of Dermatology, Dermatology Hospital of Fuzhou First General Hospital, Fujian Medical University, Fuzhou, Fujian, China; 2 Department of Dermatology, Fujian Provincial Geriatric Hospital, Fuzhou, Fujian, China

**Keywords:** keratin 16, pachyonychia congenita, palmoplantar epidermal differentiation disorder, pediatric, simvastatin, topical therapy

## Abstract

Palmoplantar epidermal differentiation disorder associated with pachyonychia congenita is a rare autosomal dominant genodermatosis characterized by painful palmoplantar hyperkeratosis and nail dystrophy. Treatment options remain limited, particularly in young children, in whom systemic therapies carry safety concerns. Herein, we report a 2-year-old girl who presented with palmoplantar hyperkeratosis since 1 month of age, with progressive nail dystrophy involving all 20 nails. Whole-exome sequencing identified a hemizygous multi-exon deletion in keratin 16 (ClinVar: SUB16259296; OMIM: #167200), and orthogonal validation using amplicon-based high-throughput sequencing confirmed a 437-nucleotide deletion predicted to result in a frameshift with premature termination codon (PTC). American College of Medical Genetics and Genomics classification supported likely pathogenicity. Twice-daily application of topical 2.5% simvastatin/cholesterol cream for 11 weeks produced substantial improvement in plantar hyperkeratosis, resolution of painful fissures, and visibly improved morphology of nascent nail growth. No local or systemic adverse effects were observed. This case provides preliminary evidence that topical simvastatin may be an effective and well-tolerated therapy for keratin 16-associated palmoplantar epidermal differentiation disorder in young children, and highlights the potential importance of topical drug delivery in achieving therapeutic efficacy for this genetic subtype.

## 1 Introduction

Palmoplantar epidermal differentiation disorders are inherited keratinization disorders affecting the palms and soles. Pachyonychia congenita, the prototypic disorder, is a rare autosomal dominant condition characterized by palmoplantar keratoderma, nail dystrophy, and plantar pain (1). Prevalence is estimated at between 1 in 10,000 and 1 in 50,000, with most individuals presenting before 10 years of age (2). The disease profoundly impacts quality of life, with plantar pain frequently limiting ambulation and disrupting sleep in pediatric patients (2).

In 2025, Sprecher et al. proposed a pathogenesis-based classification incorporating the causative gene into disease nomenclature, designating cases as palmoplantar epidermal differentiation disorder associated with pachyonychia congenita (1). This framework groups disorders caused by variants in keratin 6A, 6B, 6C, 16, and 17, acknowledging shared disruptions in keratin filament assembly while recognizing genotype-specific differences (1). Variants in keratin 16 are associated with nail dystrophy, palmoplantar keratoderma, and frequently oral leukokeratosis and cystic lesions (1).

The pathophysiology centers on dominant-negative or haploinsufficient effects of mutant keratins that disrupt keratinocyte cytoskeletal integrity and trigger inflammatory cascades (3, 4). Affected keratinocytes additionally demonstrate increased oxidative stress and diminished keap1-nuclear factor erythroid 2-related factor 2 (Nrf2) pathway activity (4). Treatment options remain largely supportive, including emollients, keratolytics such as urea, and mechanical debridement (3, 4). Systemic retinoids provide partial benefit but are poorly tolerated in children (3). Topical therapies achieving adequate local drug concentrations while limiting systemic absorption would be particularly valuable, yet few such options have been evaluated.

Statins have emerged as a candidate therapy following the demonstration that simvastatin downregulates keratin 6A promoter activity (5). Oral statins have shown benefit in keratin 6A-associated disease (6–9), but recent data suggest limited efficacy with systemic administration in keratin 16-associated or keratin 17-associated disease (10). Whether topical statin delivery might overcome this genotype-dependent differential response remains unexplored. Herein, we describe a 2-year-old girl with keratin 16-associated disease caused by a hemizygous multi-exon loss-of-function deletion who achieved substantial improvement with topical 2.5% simvastatin/cholesterol cream.

## 2 Case report

### 2.1 History and clinical findings

A 2-year-old girl of Han Chinese ethnicity presented to the dermatology clinic with a history of palmoplantar keratoderma first noted at 1 month of age. The hyperkeratosis was most pronounced over-weight-bearing areas of the plantar surfaces and periungual regions, and was accompanied by painful plantar fissures that limited ambulation. Fungal microscopy of skin scrapings was repeatedly negative. Initial treatment with topical urea and dexamethasone cream produced slight improvement. However, the condition gradually worsened over the subsequent months.

**Figure 1:**
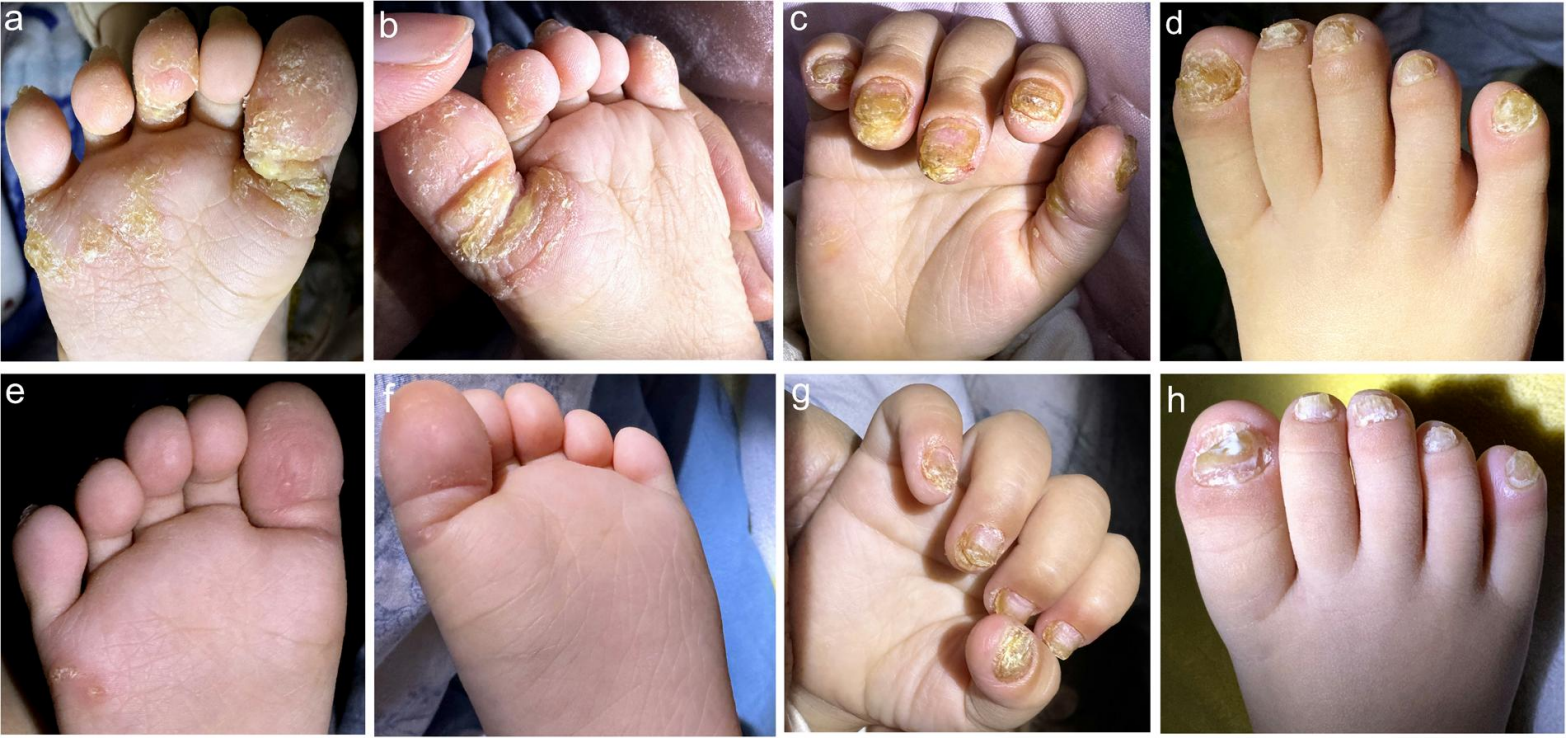
Clinical imaging before **(a–d)** and after 11 weeks of treatment **(e–h)** with 2.5% simvastatin/cholesterol cream in a 2-year-old girl with keratin 16-associated palmoplantar epidermal differentiation disorder. **(a,b)** Hyperkeratotic plaques and painful fissures on the plantar aspect of both feet. **(c,d)** Marked nail thickening and yellow-brown discoloration. **(e,f)** Plantar plaques substantially subsided and fissures healed. **(g,h)** Nascent nail growth was visibly thinner with improved morphology.

By the time of presentation, all 20 nails showed marked thickening with yellow-brown discoloration. The patient had developed natal teeth at 9 months of age and perioral and perianal keratoderma by 1 year. Physical examination revealed diffuse symmetric palmoplantar keratoderma with well-demarcated hyperkeratotic plaques, painful fissures on the plantar aspect of both feet, and thickened, dystrophic nails (Figures 1a–d). The plantar keratoderma was most prominent over the heels and metatarsal heads, where deep painful fissures interfered with walking. The fissures measured approximately 2–5 mm in depth and were associated with mild bleeding upon weight-bearing. The patient exhibited a toe-walking gait to avoid pressure on the heel fissures. Nail examination revealed thickened nail plates with subungual hyperkeratosis, onycholysis of the distal nail bed, and severe nail ridging. The oral mucosa showed mild leukokeratosis on the bilateral buccal surfaces. Her parents were non-consanguineous, clinically unaffected, and reported no family history of similar skin or nail abnormalities. The family resides in an urban area of Fujian Province, China, with adequate access to healthcare. The parents reported significant psychosocial stress related to the child's chronic pain, sleep disturbance, and limited mobility, which affected the family's daily routines and quality of life.

Prior topical therapies comprised: (i) 10% urea cream applied twice daily for 4 weeks beginning at age 3 months, yielding minimal keratolytic effect without fissure resolution; (ii) 0.05% dexamethasone cream applied twice daily for 2 weeks beginning at age 5 months, resulting in modest erythema reduction without keratoderma improvement; and (iii) petrolatum-based emollient therapy administered continuously, which afforded transient symptomatic relief but produced no objective therapeutic benefit. Systemic retinoid and immunosuppressive therapies were withheld because of the patient's young age and parental refusal due to concerns regarding developmental toxicity.

### 2.2 Genetic analysis

Whole-exome sequencing identified a hemizygous deletion within the keratin 16 gene (NM_005557.4). Due to inherent limitations of whole-exome sequencing in accurately defining large structural variants, orthogonal validation was performed using amplicon-based high-throughput sequencing. Sequence alignment confirmed a hemizygous deletion. Following Human Genome Variation Society recommendations, the variant is designated as NM_005557.4:c.617_1053del, NC_000017.11:g.41610860_41611499del (GRCh38), p.Gly206Glufs*14, predicted to result in a frameshift with premature termination codon (PTC) after 14 altered amino acids. This deletion removes 437 coding nucleotides between exons 4 and 5, encompassing a substantial portion of the central rod domain of the keratin 16 protein. Classification according to American College of Medical Genetics and Genomics guidelines supports likely pathogenic status. Parental gene sequencing was performed to determine the inheritance pattern. Neither the father nor the mother carried the KRT16 mutation variant, confirming that the deletion arose *de novo*. Representative report from parental blood samples are provided in the Supplementary Material.

### 2.3 Treatment and outcome

Given the patient's young age and concerns regarding systemic toxicity with oral agents, a compounded topical 2.5% simvastatin/cholesterol cream was prepared by the Dermatology Hospital of Southern Medical University pharmacy department. The formulation contained 2% simvastatin, 2.5% cholesterol (as a vehicle and permeation enhancer), white petrolatum, and purified water. No dimethyl sulfoxide (DMSO) or additional active ingredients were included. The cream was applied twice daily to all affected skin and nail surfaces. The parents were instructed to apply a thin layer (approximately 0.1 mm) to the plantar surfaces, periungual skin, and nail plates, gently rubbing until fully absorbed. Treatment was administered after morning bathing and before bedtime. No occlusion was used. The parents were provided with a treatment diary to record daily application, skin appearance, and any adverse events. No changes in the therapeutic regimen were required during the 11-week treatment period.

After 11 weeks of treatment, the palmoplantar keratoderma was substantially improved, all plantar fissures had healed, and nascent nail growth was visibly thinner with improved morphology (Figures 1e–h). The child was able to walk comfortably for the first time without apparent pain. Her mother reported dramatically improved sleep quality and markedly reduced distress during daily activities. Clinician-assessed outcomes at the 11-week follow-up visit included: resolution of plantar fissures (confirmed by physical examination), reduction in hyperkeratotic plaque thickness by approximately 70% (estimated clinically), and improved nail plate morphology with reduced subungual hyperkeratosis. Patient/parent-assessed outcomes included: elimination of pain during ambulation, restoration of normal sleep pattern, and return to age-appropriate play activities.

Intervention adherence was evaluated via parental follow-up interviews, who reported an adherence rate of >95% for the twice-daily dosing regimen. Tolerability was assessed by directly inquiring about local symptoms (burning, itching) and clinical examination for dermatitis, and no intolerance was reported.

## 3 Discussion

The 2025 reclassification by Sprecher et al. (1) represents a conceptual shift toward pathogenesis-based nomenclature that carries direct therapeutic implications. By incorporating the causative keratin gene into disease designation, this framework acknowledges that genotype may be a determinant of treatment response, a hypothesis increasingly supported by emerging clinical data.

The primary cause of these disorders is heterozygous variants in keratin 6A, keratin 6B, keratin 6C, keratin 16, or keratin 17, which produce structural defects in keratin filaments and aberrant keratinocyte differentiation (3, 4). Affected keratinocytes additionally demonstrate increased oxidative stress and diminished keap1-Nrf2 pathway activity, contributing to chronic sterile inflammation and hyperproliferation (4). These insights have catalyzed interest in repurposing statins as potential disease-modifying agents.

The rationale for statin therapy derives from the work of Zhao et al. (5), who demonstrated that simvastatin downregulates keratin 6A promoter activity and suppresses keratinocyte hyperproliferation in preclinical models, providing a plausible basis for clinical benefit. Subsequent case reports documented successful treatment with oral statins, predominantly rosuvastatin, in patients with keratin 6A-associated disease (6, 7), and Iqneibi et al. (8) reported benefit with oral simvastatin in a patient with a keratin 6A variant. Theocharopoulos and O'Toole (9) noted the disease-modifying potential of statins in an accompanying editorial. However, Itamoto et al. (10) recently reported that oral statin therapy was ineffective in six patients with keratin 16-associated or keratin 17-associated disease, all of whom discontinued treatment due to lack of perceived benefit. Interestingly, our findings differ from those reported by Itamoto et al., who observed no apparent clinical benefit from oral statin therapy in patients with KRT16- and KRT17-associated disease (10). The reasons for this discrepancy remain uncertain. Potential explanations include differences in drug delivery route, formulation characteristics, local tissue exposure, patient age, genetic background, and disease phenotype. In particular, topical administration may allow higher local drug concentrations at the site of pathology while limiting systemic exposure, although this hypothesis requires further investigation.

The present case is notable for several reasons. First, to our knowledge, this represents the first reported case of successful topical simvastatin therapy in keratin 16-associated disease. The marked improvement stands in direct contrast to the negative outcomes with oral statins in the keratin 16 and keratin 17 cohort reported by Itamoto et al. (10), suggesting that route of administration may be an important factor contributing to the observed efficacy. However, we acknowledge that the specific formulation, including the cholesterol vehicle and the compounding base, may also contribute to therapeutic efficacy. Cholesterol may enhance transdermal penetration and simultaneously support barrier repair, potentially offering additive benefit beyond the pharmacological action of simvastatin alone. Topical delivery achieves high local drug concentrations at the site of pathology while minimizing systemic exposure, circumventing hepatic first-pass metabolism and plasma protein binding that may constrain oral therapy. Second, this case demonstrates both efficacy and favorable safety in a 2-year-old child. The absence of local irritation or systemic adverse effects over 11 weeks supports the tolerability of topical simvastatin in toddlers, for whom systemic statin therapy carries concerns related to developmental toxicity and long-term metabolic effects. Third, the response in a patient harboring a multi-exon loss-of-function deletion suggests that statins may be beneficial even in the context of haploinsufficiency rather than dominant-negative missense variants. This observation broadens therapeutic applicability beyond patients with point mutations, suggesting that downstream mechanisms, including suppression of wild-type keratin expression, modulation of mevalonate pathway-dependent inflammation, activation of the keap1-Nrf2 antioxidant response, or restoration of barrier lipids via the cholesterol vehicle, may contribute to efficacy.

The differential response between rapidly improving cutaneous hyperkeratosis and slower nail matrix improvement is consistent with intrinsic tissue kinetics: epidermal turnover proceeds over days to weeks, whereas nail plate growth requires months to manifest structural changes. This temporal dissociation has implications for trial design and patient counseling, as nail response may continue to improve beyond the observation period of cutaneous benefit.

Certainly, this report has several limitations. As a single-case observational study, it lacks a control group and randomization. The possibility of spontaneous disease fluctuation, seasonal variation, or placebo effect cannot be excluded. The absence of a washout period or crossover design limits causal inference. The follow-up period of 11 weeks, while sufficient to observe cutaneous benefits, is insufficient to evaluate long-term nail matrix remodeling, sustained remission, or the occurrence of tachyphylaxis. Moreover, the generalizability of these findings is uncertain: the patient harbored a specific multi-exon deletion, and responses may differ in patients with missense variants, splice-site mutations, or other KRT16 alterations. Similarly, efficacy in other age groups, ethnic populations, or with alternative statin formulations (e.g., different concentrations, vehicles, or statin types) remains to be determined. Strengths of this report include the use of orthogonal genetic validation (whole-exome sequencing plus amplicon-based sequencing), objective photographic documentation, and systematic safety monitoring with laboratory parameters.

## 4 Conclusion

This case provides preliminary evidence that topical 2.5% simvastatin/cholesterol cream may be an effective and well-tolerated therapy for keratin 16-associated palmoplantar epidermal differentiation disorder in young children. The favorable response, in contrast to recent negative oral statin data in the same genetic subtype, highlights the potential importance of drug delivery route and formulation as a determinant of efficacy in genotype-directed therapy. These observations warrant validation in larger controlled trials with pediatric-appropriate endpoints.

## Data Availability

The original contributions presented in the study are publicly available. This data can be found here: https://www.ncbi.nlm.nih.gov/clinvar/variation/4856814/.
