# Supplementary material for: Case Report: Successful topical simvastatin therapy in a 2-year-old girl with keratin 16-associated palmoplantar epidermal differentiation disorder
Source: Front Pediatr. 2026 Jul 2;14:1891570. doi: 10.3389/fped.2026.1891570 (PMC13373951; doi:10.3389/fped.2026.1891570)
Supplement: Supplementary file 1 [file Datasheet1.pdf]

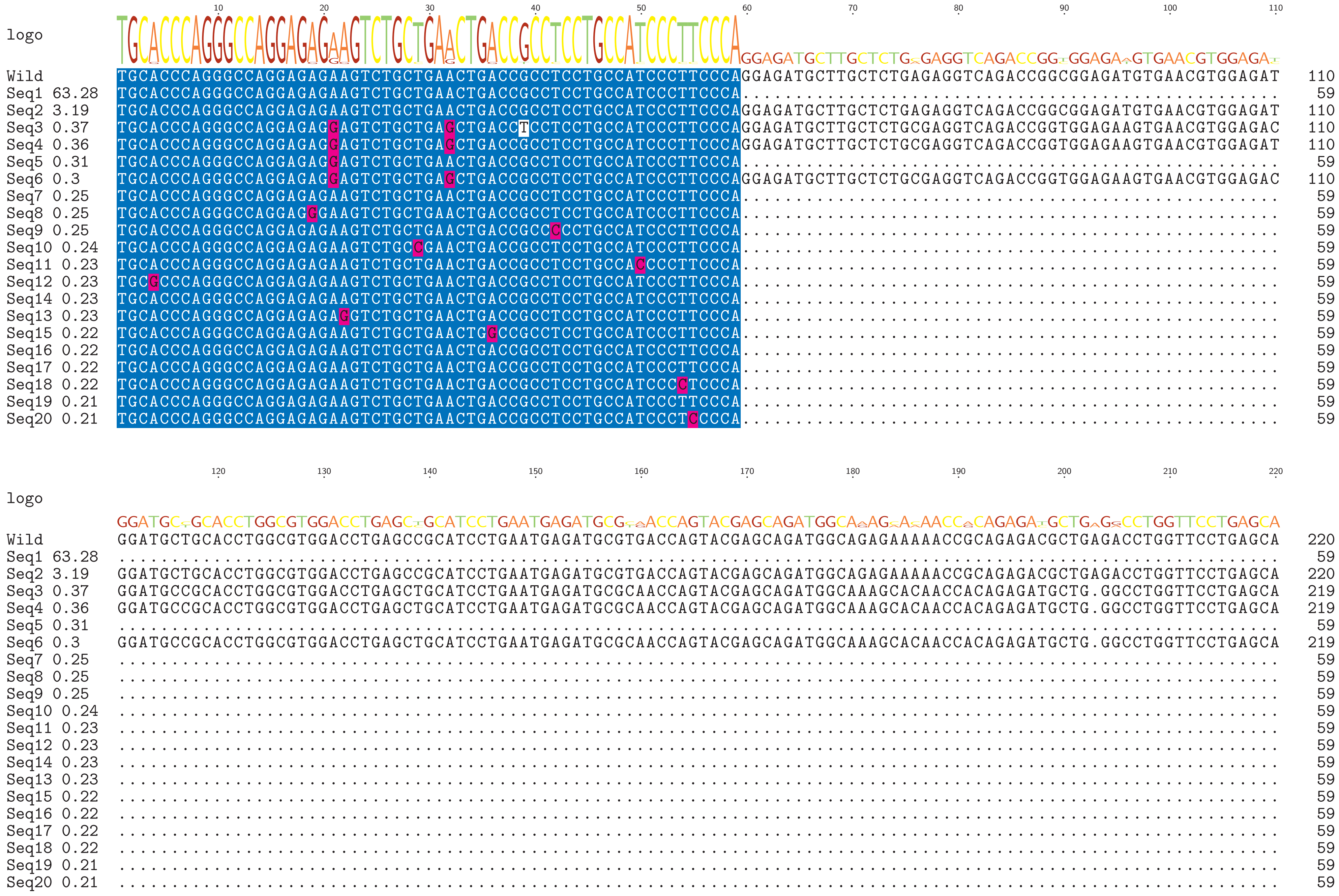
logo
Wild TGCACCCAGGGCCAGGAGAGAAGTCTGCTGAACTGACCGCCTCCTGCCATCCCTTCCCAGGAGATGCTTGCTCTGAGAGGTCAGACCGGCGGAGATGTGAACGTGGAGAT 110
Seq1 63.28 TGCACCCAGGGCCAGGAGAGAAGTCTGCTGAACTGACCGCCTCCTGCCATCCCTTCCCA... 59
Seq2 3.19 TGCACCCAGGGCCAGGAGAGAAGTCTGCTGAACTGACCGCCTCCTGCCATCCCTTCCCAGGAGATGCTTGCTCTGAGAGGTCAGACCGGCGGAGATGTGAACGTGGAGAT 110
Seq3 0.37 TGCACCCAGGGCCAGGAGAGGAGTCTGCTGAGCTGACCTCCTCCTGCCATCCCTTCCCAGGAGATGCTTGCTCTGCGAGGTCAGACCGGTGGAGAAGTGAACGTGGAGAC 110
Seq4 0.36 TGCACCCAGGGCCAGGAGAGGAGTCTGCTGAGCTGACCGCCTCCTGCCATCCCTTCCCAGGAGATGCTTGCTCTGCGAGGTCAGACCGGTGGAGAAGTGAACGTGGAGAT 110
Seq5 0.31 TGCACCCAGGGCCAGGAGAGGAGTCTGCTGAACTGACCGCCTCCTGCCATCCCTTCCCA... 59
Seq6 0.3 TGCACCCAGGGCCAGGAGAGGAGTCTGCTGAGCTGACCGCCTCCTGCCATCCCTTCCCAGGAGATGCTTGCTCTGCGAGGTCAGACCGGTGGAGAAGTGAACGTGGAGAC 110
Seq7 0.25 TGCACCCAGGGCCAGGAGAGAAGTCTGCTGAACTGACCGCCTCCTGCCATCCCTTCCCA... 59
Seq8 0.25 TGCACCCAGGGCCAGGAGGGAAGTCTGCTGAACTGACCGCCTCCTGCCATCCCTTCCCA... 59
Seq9 0.25 TGCACCCAGGGCCAGGAGAGAAGTCTGCTGAACTGACCGCCCCCTGCCATCCCTTCCCA... 59
Seq10 0.24 TGCACCCAGGGCCAGGAGAGAAGTCTGCCGAACTGACCGCCTCCTGCCATCCCTTCCCA... 59
Seq11 0.23 TGCACCCAGGGCCAGGAGAGAAGTCTGCTGAACTGACCGCCTCCTGCCACCCCTTCCCA... 59
Seq12 0.23 TGCGCCCAGGGCCAGGAGAGAAGTCTGCTGAACTGACCGCCTCCTGCCATCCCTTCCCA... 59
Seq14 0.23 TGCACCCAGGGCCAGGAGAGAAGTCTGCTGAACTGACCGCCTCCTGCCATCCCTTCCCA... 59
Seq13 0.23 TGCACCCAGGGCCAGGAGAGAGGTCTGCTGAACTGACCGCCTCCTGCCATCCCTTCCCA... 59
Seq15 0.22 TGCACCCAGGGCCAGGAGAGAAGTCTGCTGAACTGGCCGCCTCCTGCCATCCCTTCCCA... 59
Seq16 0.22 TGCACCCAGGGCCAGGAGAGAAGTCTGCTGAACTGACCGCCTCCTGCCATCCCTTCCCA... 59
Seq17 0.22 TGCACCCAGGGCCAGGAGAGAAGTCTGCTGAACTGACCGCCTCCTGCCATCCCTTCCCA... 59
Seq18 0.22 TGCACCCAGGGCCAGGAGAGAAGTCTGCTGAACTGACCGCCTCCTGCCATCCCCTCCCA... 59
Seq19 0.21 TGCACCCAGGGCCAGGAGAGAAGTCTGCTGAACTGACCGCCTCCTGCCATCCCTTCCCA... 59
Seq20 0.21 TGCACCCAGGGCCAGGAGAGAAGTCTGCTGAACTGACCGCCTCCTGCCATCCCTCCCCA... 59
logo
Wild GGATGCTGCACCTGGCGTGGACCTGAGCCGCATCCTGAATGAGATGCGTGACCAGTACGAGCAGATGGCAGAGAAAAACCGCAGAGACGCTGAGACCTGGTTCCTGAGCA 220
Seq1 63.28 59
Seq2 3.19 GGATGCTGCACCTGGCGTGGACCTGAGCCGCATCCTGAATGAGATGCGTGACCAGTACGAGCAGATGGCAGAGAAAAACCGCAGAGACGCTGAGACCTGGTTCCTGAGCA 220
Seq3 0.37 GGATGCCGCACCTGGCGTGGACCTGAGCTGCATCCTGAATGAGATGCGCAACCAGTACGAGCAGATGGCAAAGCACAACCACAGAGATGCTG.GGCCTGGTTCCTGAGCA 219
Seq4 0.36 GGATGCCGCACCTGGCGTGGACCTGAGCTGCATCCTGAATGAGATGCGCAACCAGTACGAGCAGATGGCAAAGCACAACCACAGAGATGCTG.GGCCTGGTTCCTGAGCA 219
Seq5 0.31 59
Seq6 0.3 GGATGCCGCACCTGGCGTGGACCTGAGCTGCATCCTGAATGAGATGCGCAACCAGTACGAGCAGATGGCAAAGCACAACCACAGAGATGCTG.GGCCTGGTTCCTGAGCA 219
Seq7 0.25 59
Seq8 0.25 59
Seq9 0.25 59
Seq10 0.24 59
Seq11 0.23 59
Seq12 0.23 59
Seq14 0.23 59
Seq13 0.23 59
Seq15 0.22 59
Seq16 0.22 59
Seq17 0.22 59
Seq18 0.22 59
Seq19 0.21 59
Seq20 0.21 59

```
             230       240       250       260       270       280       290       300       310       320       330
logo
```

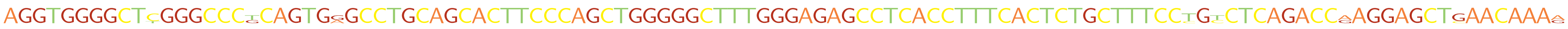

```
Wild        AGGTGGGGCTCGGGCCCGCAGTGAGCCTGCAGCACTTCCCAGCTGGGGGCTTTGGGAGAGCCTCACCTTTCACTCTGCTTTCCTGCCTCAGACCGAGGAGCTGAACAAAG  330
Seq1 63.28  ..............................................................................................................  59
Seq2 3.19   AGGTGGGGCTCGGGCCCGCAGTGAGCCTGCAGCACTTCCCAGCTGGGGGCTTTGGGAGAGCCTCACCTTTCACTCTGCTTTCCTGCCTCAGACCGAGGAGCTGAACAAAG  330
Seq3 0.37   AGGTGGGGCTCGGGCCCTCAGTGGGCCTGCAGCACTTCCCAGCTGGGGGCTTTGGGAGAGCCTCACCTTTCACTCTGCTTTCCTGTCTCAGACCAAGGAGCTCAACAAAA  329
Seq4 0.36   AGGTGGGGCTCGGGCCCTCAGTGGGCCTGCAGCACTTCCCAGCTGGGGGCTTTGGGAGAGCCTCACCTTTCACTCTGCTTTCCCGTCTCAGACCAAGGAGCTGAACAAAA  329
Seq5 0.31   ..............................................................................................................  59
Seq6 0.3    AGGTGGGGCTTGGGCCCTCAGTGGGCCTGCAGCACTTCCCAGCTGGGGGCTTTGGGAGAGCCTCACCTTTCACTCTGCTTTCCTGTCTCAGACCAAGGAGCTGAACAAAA  329
Seq7 0.25   ..............................................................................................................  59
Seq8 0.25   ..............................................................................................................  59
Seq9 0.25   ..............................................................................................................  59
Seq10 0.24  ..............................................................................................................  59
Seq11 0.23  ..............................................................................................................  59
Seq12 0.23  ..............................................................................................................  59
Seq14 0.23  ..............................................................................................................  59
Seq13 0.23  ..............................................................................................................  59
Seq15 0.22  ..............................................................................................................  59
Seq16 0.22  ..............................................................................................................  59
Seq17 0.22  ..............................................................................................................  59
Seq18 0.22  ..............................................................................................................  59
Seq19 0.21  ..............................................................................................................  59
Seq20 0.21  ..............................................................................................................  59
```

```
             340       350       360       370       380       390       400       410       420       430       440
logo
```

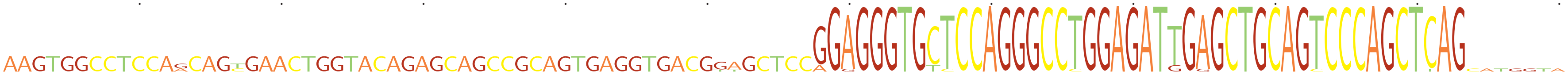

```
Wild        AAGTGGCCTCCAACAGCGAACTGGTACAGAGCAGCCGCAGTGAGGTGACGGAGCTCCGGAGGGTGCTCCAGGGCCTGGAGATTGAGCTGCAGTCCCAGCTCAGCATGGTA  440
Seq1 63.28  .........................................................GGAGGGTGCTCCAGGGCCTGGAGATTGAGCTGCAGTCCCAGCTCAG.......  105
Seq2 3.19   AAGTGGCCTCCAACAGCGAACTGGTACAGAGCAGCCGCAGTGAGGTGACGGAGCTCCGGAGGGTGCTCCAGGGCCTGGAGATTGAGCTGCAGTCCCAGCTCAG.......  433
Seq3 0.37   AAGTGGCCTCCAGCAGTGAACTGGTACAGAGCAGCCGCAGTGAGGTGACGGAGCTCCAGAGGGTGTTCCAGGGCCTGGAGATGGAGCTGCAGTCCCAGCTCAG.......  432
Seq4 0.36   AAGTGGCCTCCAGCAGTGAACTGGTACAGAGCAGCCGCAGTGAGGTGACGTTGCTCCAGAGGGTGTTCCAGGGCCTGGAGATGGAGCTGCAGTCCCAGCTTAG.......  432
Seq5 0.31   .........................................................GGAGGGTGCTCCAGGGCCTGGAGATTGAGCTGCAGTCCCAGCTCAG.......  105
Seq6 0.3    AAGTGGCCTCCAGCAGTGAACTGGTACAGAGCAGCCGCAGTGAGGTGACGGAGCTCCAGAGGGTGTTCCAGGGCCTGGAGATGGAGCTGCAGTCCCAGCTCAG.......  432
Seq7 0.25   .........................................................GGAGGGTGCTCCAGGGCCTGGAGATTGGGCTGCAGTCCCAGCTCAG.......  105
Seq8 0.25   .........................................................GGAGGGTGCTCCAGGGCCTGGAGATTGAGCTGCAGTCCCAGCTCAG.......  105
Seq9 0.25   .........................................................GGAGGGTGCTCCAGGGCCTGGAGATTGAGCTGCAGTCCCAGCTCAG.......  105
Seq10 0.24  .........................................................GGAGGGTGCTCCAGGGCCTGGAGATTGAGCTGCAGTCCCAGCTCAG.......  105
Seq11 0.23  .........................................................GGAGGGTGCTCCAGGGCCTGGAGATTGAGCTGCAGTCCCAGCTCAG.......  105
Seq12 0.23  .........................................................GGAGGGTGCTCCAGGGCCTGGAGATTGAGCTGCAGTCCCAGCTCAG.......  105
Seq14 0.23  .........................................................GGAGGGTGCTCCAGGGCCCGGAGATTGAGCTGCAGTCCCAGCTCAG.......  105
Seq13 0.23  .........................................................GGAGGGTGCTCCAGGGCCTGGAGATTGAGCTGCAGTCCCAGCTCAG.......  105
Seq15 0.22  .........................................................GGAGGGTGCTCCAGGGCCTGGAGATTGAGCTGCAGTCCCAGCTCAG.......  105
Seq16 0.22  .........................................................GGAGGGTGCCCCAGGGCCTGGAGATTGAGCTGCAGTCCCAGCTCAG.......  105
Seq17 0.22  .........................................................GGGGGGTGCTCCAGGGCCTGGAGATTGAGCTGCAGTCCCAGCTCAG.......  105
Seq18 0.22  .........................................................GGAGGGTGCTCCAGGGCCTGGAGATTGAGCTGCAGTCCCAGCTCAG.......  105
Seq19 0.21  .........................................................GGAGGGTGCTCCAGGGCCTGGAGATTGAGCTGCAGCCCCAGCTCAG.......  105
Seq20 0.21  .........................................................GGAGGGTGCTCCAGGGCCTGGAGATTGAGCTGCAGTCCCAGCTCAG.......  105
```

```
                        450
logo

              TGAAGGACCCAGCA
Wild          TGAAGGACCCAGCA   454
Seq1 63.28    ..............   105
Seq2 3.19     ..............   433
Seq3 0.37     ..............   432
Seq4 0.36     ..............   432
Seq5 0.31     ..............   105
Seq6 0.3      ..............   432
Seq7 0.25     ..............   105
Seq8 0.25     ..............   105
Seq9 0.25     ..............   105
Seq10 0.24    ..............   105
Seq11 0.23    ..............   105
Seq12 0.23    ..............   105
Seq14 0.23    ..............   105
Seq13 0.23    ..............   105
Seq15 0.22    ..............   105
Seq16 0.22    ..............   105
Seq17 0.22    ..............   105
Seq18 0.22    ..............   105
Seq19 0.21    ..............   105
Seq20 0.21    ..............   105
```
