# Supplementary material for: Case Report: Successful topical simvastatin therapy in a 2-year-old girl with keratin 16-associated palmoplantar epidermal differentiation disorder
Source: Front Pediatr. 2026 Jul 2;14:1891570. doi: 10.3389/fped.2026.1891570 (PMC13373951; doi:10.3389/fped.2026.1891570)
Supplement: Supplementary file 2 [file Datasheet2.pdf]

Data Sheet 2: Legend for Sequence Data

Amplicon-based High-Throughput Sequencing Validation of KRT16 (NM_005557.4) c.617_1053del

1. Methodology

1.1 Library Preparation and Sequencing

Specific PCR products were sent to Sangon Biotech (Shanghai, China) Co., Ltd. for sequencing library preparation and sequencing. Library preparation was performed by two-step PCR. First-round PCR reaction was set up as follows: DNA template (10 ng/μl) 2 μl; amplicon PCR forward primer mix (10 μM) 1 μl; amplicon PCR reverse primer mix (10 μM) 1 μl; 2× PCR Ready Mix 15 μl (total 25 μl) (Kapa HiFi Ready Mix). The plate was sealed and PCR performed in a thermal instrument (BIO-RAD, T100TM) using the following program: 1 cycle of denaturing at 98 °C for 3 min, then 8 cycles of denaturing at 98 °C for 30 s, annealing at 60 °C for 30 s, elongation at 72 °C for 30 s, and a final extension at 72 °C for 5 min. The PCR products were checked using electrophoresis in 2 % (w/v) agarose gels and purified using AMPure XP magnetic beads. The second-round PCR was performed to obtain sequencing libraries with molecular tags. The reaction was set up as follows: DNA template (10 ng/μl) 2 μl; universal P7 primer with index (10 μM) 1 μl; P5 primer with index (10 μM) 1 μl; 2× PCR Ready Mix 15 μl (total 30 μl) (Kapa HiFi Ready Mix). The program was: 1 cycle of denaturing at 98 °C for 5 min, then 5 cycles of denaturing at 94 °C for 30 s, annealing at 55 °C for 20 s, elongation at 72 °C for 30 s, and a final extension at 72 °C for 5 min. The final PCR products were purified using AMPure XP magnetic beads. The libraries were quantified and pooled. Paired-end sequencing was performed on the Nextseq2000 sequencer (Illumina, San Diego, CA) with PE300 model.

1.2 Data Quality Control and Analysis

Raw reads were filtered according to the following steps: (a) adaptor sequences were removed using Cutadapt (v1.2.1) with default parameters; (b) low-quality bases were removed from reads 3' to 5' ($Q < 20$) using PRINSEQ-lite (v0.19.5) with a sliding window approach (window size = 5, length threshold = 50); (c) chimeric sequences were removed using USEARCH (v11.0.667) with de novo mode and default parameters; (d) paired-end reads were merged using PEAR (v0.9.6); (e) target sequences were extracted using a custom Python script when both forward and reverse primers perfectly matched; (f) sequence frequency was calculated and the top 20 most frequent unique sequences were selected for downstream analysis.

Quality control metrics:

• Total raw reads: 195,224 (R1) / 195,224 (R2)
• Total bases: 58,762,424 bp
• Post-QC reads: 190,288
• Post-QC bases: 42,362,620 bp
• Average length after QC: 222.62 bp

• Q20: 98.75%
• Q30: 95.39%
• Non-chimeric reads: 190,288 (100% of post-QC reads)

1.3 Sequence Alignment and Visualization

The top 20 most frequent unique sequences were aligned with the wild-type reference sequence using MUSCLE (v3.8.31). Sequence alignment visualization was generated using a custom R script (R v4.1.0). The reference sequence (designated "Wild") was derived from the human genome assembly GRCh38 (NC_000017.11).

2. Figure Description

The figure displays aligned segments of the amplicon. Each panel contains the following components:

- "**logo**": Sequence logo representing nucleotide conservation across all aligned reads. The height of each letter reflects the relative frequency of that nucleotide at the given position.
- "**Wild**": The reference (wild-type) sequence derived from the human genome assembly GRCh38 (NC_000017.11).
- "**Seq1–Seq20**": The top 20 representative sequencing reads from the patient sample, selected by alignment quality and frequency. The numeric value to the left of each sequence ID indicates the relative frequency (percentage) of that unique sequence in the total effective reads. For example, "Seq1 63.28" indicates that this unique sequence accounts for 63.28% of all effective reads. The number to the right indicates the sequence length in base pairs.

3. Mutation Detection Results & Mutation Analysis

3.1 Top 20 Sequence Mutations Overview

The following table presents detailed mutation information for the top 20 sequences. Wild-type (Wild) serves as the reference, with Seq1–Seq20 ranked by frequency.

Seq	%	Len	Mutation Description
Wild	-	454	Reference
Seq1	63.28	105	Large deletion at ~59 bp
Seq2	3.19	433	Wild-type match
Seq3	0.37	432	A19G,A23G,C26T,A30G,C38T
Seq4	0.36	432	Like Seq3, different at end
Seq5	0.31	105	Deletion at ~59 bp
Seq6	0.30	432	Highly similar to Seq3

Seq	%	Len	Mutation Description
Seq7-20	0.21-0.25	105	Various point mutations

3.2 Key Observations

3.2.1 The predominant allele carries a large deletion(63.28%)

Seq1, representing 63.28% of valid reads, matches the wild-type reference only for the first 59 bp and positions 388–433 bp, with complete absence of sequence from position 60 to 387. This indicates a large fragment deletion within the KRT16 target amplicon region on the patient's predominant mutant allele, resulting in severe protein truncation.

3.2.2 Low-level wild-type allele retention (3.19%)

Seq2 (3.19%) is fully matched to the wild-type reference sequence, suggesting retention of a small fraction of functional wild-type KRT16 alleles. The relatively low proportion may reflect amplification bias, differential amplification efficiency between alleles, or somatic mosaicism.

3.2.3 Scattered point mutation alleles (~1.03%)

Seq3/4/6, collectively ~1.03%, harbor multiple base substitutions (A19G, A23G, C26T, A30G, C38T, etc.) with coverage extending to 432 bp. These sequences may represent secondary pathogenic mutations or somatic mutation events.

4. Functional Impact Prediction

Predicted mechanism: The large deletion causes premature translational termination or produces severely truncated protein products incapable of forming normal intermediate filament dimers. The residual wild-type allele (~3.19%) may be insufficient to compensate for the mutant allele's loss of function, resulting in either a dominant-negative effect or haploinsufficiency.
